# Interaction between genetic regions responsible for the starch properties in non-glutinous rice varieties in Hokkaido, Japan

**DOI:** 10.1270/jsbbs.23087

**Published:** 2024-03-22

**Authors:** Tomohito Ikegaya

**Affiliations:** 1 Institute of Crop Science, National Agricultural and Food Research Organization, 2-1-18 Kannondai, Tsukuba, Ibaraki 305-8518, Japan

**Keywords:** amylose content, pasting temperature, amylopectin chain-length distribution, *Starch branching enzyme IIb*, *qAC9.3*

## Abstract

Starch properties are the major determinants of grain quality and food characteristics in rice (*Oryza sativa* L.). Understanding the interactions between genetic regions responsible for starch properties will lead to the development of rice cultivars with desirable characteristics. This study investigated the genetic effect and interaction between *qAC9.3*, a low-amylose quantitative trait locus (QTL), and the genetic region around *Starch branching enzyme IIb* (*SbeIIb*). Both these factors are responsible for the starch properties of the Hokkaido breeding population. The amylose content, pasting temperature, and amylopectin chain-length distribution were compared using F_5_ lines derived from the cross between the lower amylose content and lower pasting temperature strain ‘Hokkai332 (*qAC9.3*, *SbeIIb*)’ and the higher amylose content and higher pasting temperature variety ‘Kitagenki (-, *SbeIIb^sr^*)’. The *qAC9.3* genotype exhibited low amylose content and reduced the hardness of boiled rice but increased the ratio of amylopectin long chains and did not alter the pasting temperature. In contrast, the *SbeIIb* genotype was associated with pasting temperature but did not affect the amylose content and hardness of boiled rice. It was suggested that appropriately selecting genotypes of these genetic regions and QTL would allow the fine-tuning of starch properties of cooked rice suitable for future demand.

## Introduction

Starch properties are the major determinants of grain quality and food characteristics in rice (*Oryza sativa* L.). Amylopectin, which has a highly branched structure, is the major component of starch, whereas the remainder comprises amylose, a linear polymer. In rice, the amylose content (AC) and chain-length distribution of amylopectin branches determine the physicochemical properties of starch and the cooking properties of grains through changes in gelatinisation and starch retrogradation. The rice Waxy (*Wx*) gene (LOC_Os06g04200) encodes *granule-bound starch synthase I* (*GBSSI*), which determines the AC of the endosperm by controlling amylose synthesis (Sano 1984). Natural variations in genotypes such as *Wx-a*, *Wx-b*, and *wx* have been identified as *Wx* alleles. The AC greatly affects the hardness of cooked rice. A higher AC increases the hardness, whereas a lower AC reduces the hardness. In Japan, a soft texture is considered to indicate good eating quality. In Japan, the taste of rice has been improved by reducing AC through breeding programs (Inatsu 1988). *Wx-mq*, *Wx1-1*, and *Wx-y* alleles that exhibit low AC have been generated and selected through breeding programs (Ando *et al.* 2010, Chuba *et al.* 2006, Sato *et al.* 2002). In addition to the *Wx* allele, *dull* (*du1-5*), *qAC2*, and *qAC9.3* are genes/quantitative trait loci (QTLs) for controlling the AC in rice (Ando *et al.* 2010, Satoh and Omura 1981, 1986, Takemoto-Kuno *et al.* 2015, Yano *et al.* 1988). Some dull mutants induced by N-methyl-N-nitrosourea solution have already been analysed, and the causal genes were identified as *Du1* (Zeng *et al.* 2007) and *Du3* (Isshiki *et al.* 2008). *qAC2* and *qAC9.3* are considered natural mutations selected in rice breeding programs. The AC of a near-isogenic line carrying *qAC2^Kuiku^*, the Kuiku162 allele of *qAC2*, was 1.1% points lower than that of the wild type in the genetic background of a japonica cultivar. In addition, *qAC2^Kuiku^* has an epistatic interaction with the *Wx* allele and *du1-3* (Takemoto-Kuno *et al.* 2015). The *qAC9.3*, which causes a 2.6% decrease in AC, could be useful for fine-tuning AC in rice breeding programs (Ando *et al.* 2010). However, it was unclear whether there was any differentiation in the pasting temperature and amylopectin chain-length distribution related to starch properties and how *qAC9.3* interacts with other genes responsible for starch properties.

Starch-branching enzymes (BEs) generate amylopectin branches. Plants have two types of BE, *BEI* and *BEII*. Mutant *BEI*, *BEIIa*, and *BEIIb* lines have been isolated from rice (Nakamura 2002, Nishi *et al.* 2001, Satoh *et al.* 2003a, 2003b). Previously, *starch branching enzyme IIb* (*SbeIIb*) was identified on chromosome 2 as a candidate gene for the differences in starch properties between glutinous rice varieties in Hokkaido (Ikegaya and Ashida 2021). *SbeIIb* is known as an *amylose extender* (*AE*) gene. The *sbeIIb* mutant showed a decreased proportion of the amylopectin short chain (degree of polymerisation [DP] < 17) and an increased proportion of the mid-length chain (DP 18–35) (Nishi *et al.* 2001), resulting in a higher gelatinisation temperature (Jane *et al.* 1999). The glutinous variety/line with *SbeIIb^sr^*, the genotype originating from the American variety ‘Cody’, showed a higher pasting temperature (PT) and higher hardness of rice flour paste under the condition of storage at 4°C for 24 h (Ikegaya and Ashida 2021). The chromosomal region, including the *SbeIIb^sr^* genotype, causes changes in the chain-length distribution of amylopectin; the proportions of short (DP 6–24) and long chains (DP 25–60) decreased and increased, respectively. The *SbeIIb^sr^* genotype has been introduced into almost all elite non-glutinous rice cultivars in Hokkaido through a breeding program. However, the effect of the *SbeIIb^sr^* genotype on non-glutinous rice varieties/lines is unknown.

*qAC9.3* and *SbeIIb^sr^* are unique genotypes that characterise starch properties in the Hokkaido breeding strain; however, no genetic interactions between these genotypes have been reported. In this study, the non-glutinous rice cultivar ‘Kitagenki’ and breeding strain ‘Hokkai332’ developed in Hokkaido were selected. The cultivar ‘Kitagenki’, which has a *SbeIIb^sr^* genotype, showed extremely high yield potential; however, the eating quality of cooked rice was reduced because of its high AC without *qAC9.3*. (Ikegaya *et al.* 2017, Yagioka *et al.* 2021). Breeding strain ‘Hokkai332’, which contains *qAC9.3* and *SbeIIb*, showed a low AC and good eating quality. The effect of the *SbeIIb^sr^* genotype in non-glutinous rice varieties was characterized, and the effect of *qAC9.3* on the amylopectin chain-length distribution ratio was investigated by analysing F_5_ progenies derived from the cross between ‘Kitagenki’ and ‘Hokkai332’. Furthermore, the genetic interaction between the *SbeIIb* genotype and *qAC9.3* was evaluated for starch properties. This knowledge may contribute to meeting future demands for breeding varieties with finely tuned starch characteristics.

## Materials and Methods

### Plant material

The high-yield cultivar ‘Kitagenki’ and breeding strain ‘Hokkai332’ were used as the parental lines to detect the interaction between *qAC9.3* and the genetic regions around *SbeIIb* responsible for starch properties. F_5_ lines (n = 94), derived from a cross between ‘Kitagenki’ and ‘Hokkai332’, were investigated in 2020. Sixteen plants from each F_5_ line were grown and harvested.

### Growth conditions

Cultivation management followed the standard procedures used at the Hokkaido Agricultural Research Center as described by Ikegaya and Ashida (2021). Sowing and transplanting were performed on 20 April and 20 May 2020, respectively. The young leaves were sampled for DNA extraction. Seeds were harvested at the full maturity stage.

### Re-sequencing and DNA analysis

Isolation of total DNA and paired-end sequencing using Illumina HiSeq 4000 was performed according to Ikegaya and Ashida (2021). Raw sequence data of ‘Kitagenki’ and ‘Hokkai332’ were deposited in the DDBJ Sequence Read Archive under the accession number DRA017497. Single-nucleotide polymorphism (SNP) markers showing polymorphisms between the parental lines were selected by comparing whole-genome sequences, and genotypes were detected using the Fluidigm EP1 System (https://dnatech.genomecenter.ucdavis.edu/fluidigm-ep1/). A total of 189 SNP markers used at the Advanced Genomics Breeding Section of the Institute of Crop Science, NARO (NICS), covering all 12 chromosomes, were tested, and 166 SNP markers were selected. The single-sequence repeat marker RM23804 was used for genotyping *qAC9.3*, according to Ando *et al.* (2010). The CAPS marker SbeIIb Ex3-1 and restriction enzyme BspT107I (HgiC I) were used according to Ikegaya and Ashida (2021) to determine the genotype of the chromosomal region around *SbeIIb*.

### Detection of QTLs

QTL analysis was performed as described in Ikegaya and Ashida (2021).

### Analysis of starch properties

AC was analysed according to the method described by Ando *et al.* (2010). Starch purification, analysis of chain-length distribution, and measurement of pasting properties were performed using a Rapid Visco Analyser (RVA3D+, Newport Scientific Pty Ltd., NSW, Australia), as described in Ikegaya and Ashida (2021). In this paper, apparent AC is referred to as AC.

Hardness of boiled rice: The rice was cooked using a Rapid Visco Analyser (RVA), according to Ashida-Yoshida *et al.* (2020). Boiled rice was stored with a cover at two different conditions, 30°C for 1 h and 4°C for 24 h. The hardness of boiled rice was measured using a digital force gauge and measurement stand (DST-2N and MX-500N, Imada, Toyohashi, Japan) equipped with a 20 N load cell. A single compression force-versus time program was used to compress boiled rice along the thickness at a test speed of 0.5 mm/s and then returned to its original position. The original clearance between the probe and the bottom of the RVA can in the load cell of the instrument was fixed at 8 mm; therefore, when the probe moved down, the test sample was compressed horizontally on the base to a distance of 6.0 mm. A stainless steel probe (P/5) 10 mm in diameter was used to compress the boiled rice. The test was repeated three times for the same sample, and samples were prepared three times for the parents and all F_5_ lines. The peak force was considered as the maximum compressive force/hardness.

## Results

### AC and PT

The range of days to heading for the F_5_ lines, including both parents, was 12 days from July 18 to July 29. Average temperatures for the 20 days after heading were 20.75°C at the lowest on July 21 and 21.88°C at the highest on July 29. The AC and PT of ‘Kitagenki’ and ‘Hokkai332’ were 22.9% and 72.2°C, and 16.5% and 71.2°C, respectively (Table 1). The phenotypic distributions of AC and PT in the F_5_ lines were determined (Supplemental Fig. 1). No relationship was found between days to heading and AC (Supplemental Fig. 2). Owing to a lack of materials, the AC of the seven lines could not be measured in the F_5_ lines.

### QTLs for AC and PT

In total, 189 SNP markers were used to genotype the 12 chromosomes of the F_5_ lines. Twenty-seven markers showed statistically significant segregation ratios in the chi-squared test (Supplemental Table 1). These 27 markers were excluded from subsequent analyses. QTL analysis assessed AC and PT (Table 2, Supplemental Table 1). The QTL for AC was mapped only on chromosome 9 between the SNP markers FA6423 and FA0535 in the 7.6 Mbp region, and the nearest marker FA3224 was located 96 kbp from RM23804, an SSR marker for *qAC9.3*. The QTL for PT was mapped only on chromosome 2 between the SNP markers FA1962 and FA2444 in the 5.6 Mbp region, and the nearest marker FA0803 was located 136 kbp from SbeIIb Ex3-1, a CAPS marker that determines the genotype of the chromosomal region around *SbeIIb*. The logarithm of the odds scores of the QTLs were 23.2 and 14.1 for AC and PT, respectively. The percentages of phenotypic variation explained were 74.3% and 57.7% for AC and PT, respectively. The additive effects of the ‘Hokkai332’ allele were –1.87 (%) and –0.47 (°C) for AC and PT, respectively. The ‘Hokkai332’ genotype lowered AC and PT (Table 2, Supplemental Fig. 1).

### Amylopectin chain-length distribution patterns

**Fig. 1.**
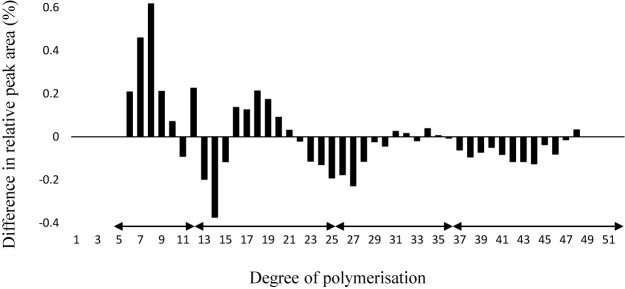
Comparison of chain-length distribution profiles of amylopectin from ‘Kitagenki’ and ‘Hokkai332’. The difference in the profiles was calculated by subtracting the ratio of a chain of a given length of ‘Kitagenki’ from that of the value of ‘Hokkai332’. Values are the means of two replicates. Black arrows indicate four groups classified by Hanashiro *et al.* (1996).

**Fig. 2.**
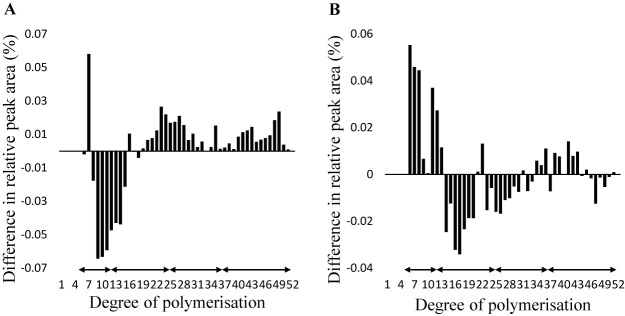
Comparison of chain-length distribution profiles of amylopectin from F_5_ lines by genotype at genetic region for starch properties. A. *qAC9.3*. B. *sbeIIb*. The difference in the profiles was calculated by subtracting the ratio of a chain of a given length of ‘Kitagenki’ from that of the value of ‘Hokkai332’. The difference in the profiles was calculated by subtracting the ratio of a chain of given length of ‘Kitagenki’ genotype from that of the ‘Hokkai332’ genotype. Values are the means of two replicates. Black arrows indicate four groups classified by Hanashiro *et al.* (1996).

The chain-length distribution was analysed to clarify the amylopectin structure of ‘Kitagenki’ and ‘Hokkai332’. The side chains were classified into four groups, according to Hanashiro *et al.* (1996). In the amylopectin chain-length distribution patterns of ‘Hokkai332’ compared to ‘Kitagenki’, the proportion of side chains with DP 6–12 was significantly increased, and the proportion of side chains with DP 25–36 and DP 37- were significantly decreased (Fig. 1, Table 3). The chain-length distribution of F_5_ lines was measured and classified according to the genotype of *qAC9.3* and *SbeIIb* (Fig. 2, Table 4). Strains that were heterozygous for the genotype of *qAC9.3* and/or *SbeIIb* were excluded from subsequent analysis. No significant difference was detected in the side chain proportions of DP 6–12 and DP 13–24 between the lines with and without *qAC9.3*. The proportions of side chains with DP 25–36 and DP 37- were significantly increased in lines with *qAC9.3* (Fig. 2a, Table 4). In lines with the genotype *SbeIIb*, the proportion of side chains with DP 6–12 was significantly higher than that in the *SbeIIb^sr^* lines. However, the proportion of side chains with DP 13–24 was significantly decreased. No significant differences were detected in the side chain proportions of DP 25–36 and DP 37- (Fig. 2b, Table 4). The F_5_ lines with the same genotype as ‘Kitagenki’ (-, *SbeIIb^sr^*) or ‘Hokkai332’ (*qAC9.3*, *SbeIIb*) showed AC and PT according to genotype, although with different patterns of parental lines in comparison of amylopectin chain-length distribution (Supplemental Fig. 2, Table 4). AC and PT also corresponded to the genotypes when comparing the genotypes (*qAC9.3*, *SbeIIb^sr^*) and genotypes (-, *SbeIIb*) that differed from those of the parental varieties.

### Hardness of boiled rice

**Fig. 3.**
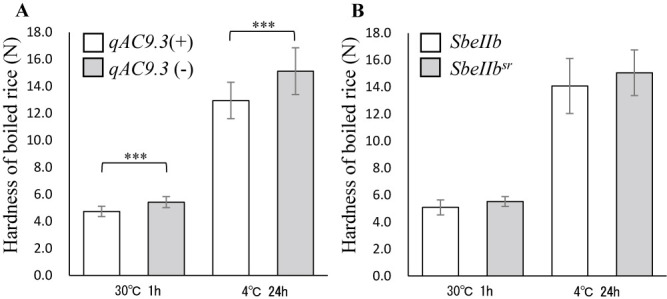
Comparison of the hardness of boiled rice between genotypes. A. Hardness of boiled rice of the *qAC9.3* genotype. *qAC9.3*(+) indicates F_5_ lines with *qAC9.3*. *qAC9.3*(–) indicates F_5_ lines without *qAC9.3*. B. Hardness of boiled rice of the *SbeIIb* genotype. * indicates a significant difference (Student’s t-test, ***P < 0.001). Error bars indicate standard deviation.

Hardness of boiled rice was markedly higher in ‘Kitagenki’ than in ‘Hokkai332’ at 30°C for 1 h and 4°C for 24 h after cooking (Table 5, Supplemental Table 1). The average hardness of F_5_ lines with *qAC9.3* (30°C: 4.7N, SD ± 0.4 and 4°C: 12.8N, SD ± 1.5) were significantly lower than lines without *qAC9.3* (30°C: 5.5N, SD ± 0.4 and 4°C: 15.0N, SD ± 1.7). Conversely, the average hardness of F_5_ lines with *SbeIIb^sr^* (30°C: 5.3N and 4°C: 14.3N) was not significantly higher than that of lines with *SbeIIb* (30°C: 5.1N and 4°C: 14.1N) (Fig. 3).

## Discussion

The breeding strain ‘Hokkai332’ shows lower AC, PT, and hardness of boiled rice than those of the high-yield cultivar ‘Kitagenki’ (Tables 1, 4). QTL analysis of F_5_ lines derived from the cross between ‘Hokkai332’ and ‘Kitagenki’ revealed that differences in AC and PT between ‘Hokkai332’ and ‘Kitagenki’ were caused by *qAC9.3* and the genetic region around *SbeIIb*, respectively. However, phenotypic values in the F_5_ population showed continuous variation (Supplemental Figs. 1, 2). The cause could be other minor genetic factors other than on the candidate region. These factors would be able to be detected more accurately by performing a multiple-year QTL analysis and phenotypic evaluation. The interaction between *qAC9.3* and the genetic region around *SbeIIb*, both of which are responsible for starch properties in rice, was investigated (Ando *et al.* 2010, Ikegaya and Ashida 2021). The average AC of the F_5_ lines with *qAC9.3* (18.2%) was lower than that of lines without *qAC9.3* (21.8%) (Table 4). In contrast, there were no differences in AC caused by the genotype of *SbeIIb* in this study (Table 4). *SbeIIb* is known as the *AE* gene. The AC of the *ae* mutant was higher than that of the wild type (Nishi *et al.* 2001). *SbeIIb^sr^*, the ‘Kitagenki’ genotype, decreased the short-chain ratio and increased the long-chain ratio in glutinous rice varieties as well as the *ae* mutant in the amylopectin chain-length distribution ratio analysis (Ikegaya and Ashida 2021). However, there was no significant effect on the AC in the glutinous rice varieties.

The average PT of F_5_ lines with *SbeIIb* (71.4°C) was lower than that of lines with *SbeIIb^sr^* (72.1°C) (Table 4). Although introducing low-amylose genotypes decreases PT (Satou *et al.* 2016, Yoshii *et al.* 1997), there was no significant difference in PT caused by the *qAC9.3* genotype (Table 4). The F_5_ lines showed different amylopectin chain-length distribution characteristics according to the genotypes of *qAC9.3* and *SbeIIb*. Significant differences were found in DP 25–36 and 37–60 between lines with and without *qAC9.3*. Although Okuno *et al.* (1983) reported no significant differences in amylopectin chain-length distribution between glutinous, low-amylose, and non-glutinous rice, differences caused by *qAC9.3* were observed. The increase in the amylopectin long-chain ratio by *qAC9.3* may mask the decrease in PT due to the low AC. In contrast, in lines with *SbeIIb*, the proportion of side chains with DP 6–12 was significantly increased compared to that in lines with *SbeIIb^sr^*. These results are the same as those of a previous report using glutinous varieties (Ikegaya and Ashida 2021). However, the proportion of side chains with DP 13–24 was significantly decreased. This chain-length distribution pattern was similar to that of *SSIIa* (Miura *et al.* 2018), *Sbe1* (Okamoto *et al.* 2013), and *Pho1* (Okamoto *et al.* 2020).

AC is known to affect the hardness of cooked rice (Yu *et al.* 2009). In this study, whether the hardness of cooked rice is affected by the *qAC9.3* genotype and the genotype of the chromosomal region around *SbeIIb* was investigated. The results showed that only the *qAC9.3* genotype and AC affected the hardness of boiled rice, and the *SbeIIb* genotype showed a slight but not significant difference in hardness. Significant differences were observed in the change in hardness of glutinous rice flour paste from lines with different *SbeIIb* genotypes when stored at 4°C for 24 h (Ikegaya and Ashida 2021); however, no differences were observed in the change in hardness of non-glutinous boiled rice. Itayagoshi *et al.* (2021) reported that AC is related to hardness but not stickiness, whereas PT is related to stickiness. This relationship between PT and stickiness is consistent with the results of previous reports (Li *et al.* 2016).

*Wx* gene expression and AC increase with the intensity of low temperatures during ripening (Hirano and Sano 1998). Under climatic conditions of low temperatures during the rice ripening period in Hokkaido, the AC tends to be high. In the Hokkaido rice breeding program, *Wx1-1* and *qAC9.3* were introduced to reduce AC and improve the taste of cooked rice (Ando *et al.* 2010). The amylopectin chain-length distribution ratio is known to increase the short-chain ratio at low temperatures during ripening (Igarashi *et al.* 2008). The reason for the introduction of *SbeIIb^sr^* into Hokkaido rice varieties remains unclear. This was possibly introduced unintentionally because selection for PT and stickiness has not been conducted in rice breeding. The distribution ratio of amylopectin short chains (DP6–12) in the lines with genotypes *qAC9.3* and *SbeIIb^sr^* was the lowest among the four genotype groups (Table 4). Both genotypes additively affected the amylopectin chain-length distribution ratio.

The AC and fine structure of amylopectin greatly affect the rice quality after cooking. Here, it was revealed that *qAC9.3* decreased AC while increasing the proportion of amylopectin long chains (DP25-) and did not change PT. This finding indicates that it was possible to fine-tune the AC of rice without changing its stickiness, which correlates with the PT. In contrast, *SbeIIb^sr^* increased PT by changing the amylopectin chain-length distribution and did not change AC. It was possible to fine-tune the PT and stickiness of rice without changing its hardness, which correlated with the AC. The *qAC9.3* and *SbeIIb^sr^* were detected in Hokkaido rice breeding lines and are expected to be used as breeding materials in different cultivation areas. The association of genotypes with useful phenotypes may be facilitated using local breeding varieties because of their close genetic backgrounds and low incidence of poor traits in the evaluations. Historical studies on the unique traits that characterise elite cultivars in each local region may contribute to developing new cultivars with novel combinations of genes and more desirable agronomic traits.

## Author Contribution Statement

Conceived and designed the experiments and wrote the manuscript: TI. Performed the experiments: TI. Analysed the data: TI.

## Supplementary Material

Supplemental Figures

Supplemental Table

## Figures and Tables

**Table 1.** Amylose content and pasting temperature of ‘Hokkai332’ and ‘Kitagenki’

Variety/Strain	AC (%)	PT (°C)	Genotype	
			*qAC9.3*	*SbeIIb*
Hokkai332	16.5	71.2	*qAC9.3*	*SbeIIb*
Kitagenki	22.9	72.2	–	*SbeIIb^sr^*

AC; amylose content. PT; pasting tempareture.

**Table 2.** QTL for amylose content and pasting temperature

Phenotype	Nearest marker	Chromosome	Position (Mbp)	LOD	Var.exp (%)	Additive effect
Amylose content	FA3224	9	1.3–8.9	23.2	74.3	–1.87 (%)
Pasting temperature	FA0803	2	16.8–22.4	14.1	57.7	–0.47 (°C)

**Table 3.** HPAEC-PAD fractions of debranched amylopectin from ‘Hokkai 332’ and ‘Kitagenki’

Variety/Strain	DP 6–12 (%)		DP 13–24 (%)	DP 25–36 (%)		DP 37–60 (%)	
Hokkai 332	32.9	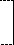 *	54.4	9.8	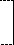 *	3.0	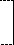 ***
Kitagenki	32.0		54.5	10.2		3.4	

* indicates significant difference (Student’s t-test, * P < 0.05, *** P < 0.001).

**Table 4.** Chain length distribution in the F_5_ lines

Genotype	Number	AC		PT		Amylopectin chain length distribution							
						DP 6–12 (%)		DP 13–24 (%)		DP 25–36 (%)		DP 37–60 (%)	
*qAC9.3*	34	18.2	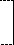 ***	71.6		32.2		54.5		10.1	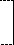 **	3.2	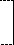 ***
–	45	21.8		71.8		32.4		54.5		10.0		3.1	
*SbeIIb*	45	20.4		71.4	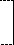 ***	32.4	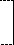 **	54.4	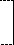 **	10.1		3.1	
*SbeIIb^sr^*	34	20.4		72.1		32.2		54.6		10.1		3.1	
*qAC9.3*, *SbeIIb*	23	18.2	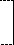 ***	71.5	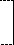 ***	32.3		54.3	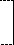 *	10.2	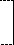 **	3.3	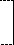 ***
-, *SbeIIb^sr^*	23	21.8		72.2		32.3		54.5		10.1		3.1	
*qAC9.3*, *SbeIIb^sr^*	11	18.3	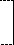 ***	72.1	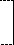 ***	32.1	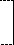 **	54.6		10.1	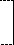 **	3.2	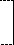 **
-, *SbeIIb*	22	21.8		71.3		32.5		54.4		10.0		3.1	

* indicates significant difference (Student’s t-test, * P < 0.05, ** P < 0.01,*** P < 0.001). ‘–’ indicates genotype without *qAC9.3*.

**Table 5.** Hardness of boiled rice of ‘Hokkai 332’ and ‘Kitagenki’

Variety/Strain	30°C 1 h (N)		4°C 24 h (N)	
Hokkai 332	4.2	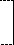 ***	11.3	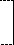 ***
Kitagenki	6.2		15.3	

* indicates significant difference (Student’s t-test, *** P < 0.001).
