# Supplementary material for: Interaction between genetic regions responsible for the starch properties in non-glutinous rice varieties in Hokkaido, Japan
Source: Breed Sci. 2024 Mar 22;74(2):159–65. doi: 10.1270/jsbbs.23087 (PMC11442103; doi:10.1270/jsbbs.23087)
Supplement: Supplementary file 1 — Supplemental Figures [file 74_159_s1.pdf]

doi: 10.1270/jsbbs.23087

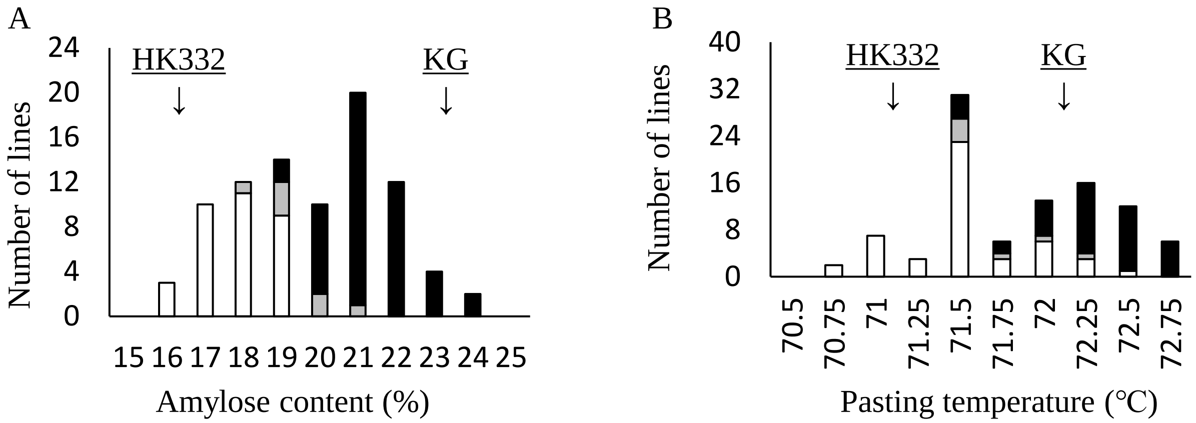

Supplemental Figure. 1. The phenotypic distributions in the F_5 lines. A. Amylose content. Black, white, and gray boxes indicate the genotypes of KG, HK332, and the heterozygous lines, respectively, in RM23804. B. Pasting temperature. Black, white, and gray boxes indicate the genotypes of KG, HK332, and the heterozygous lines, respectively, in SbeIIb Ex3-1. KG: ‘Kitagenki’, HK332: ‘Hokkai332’.

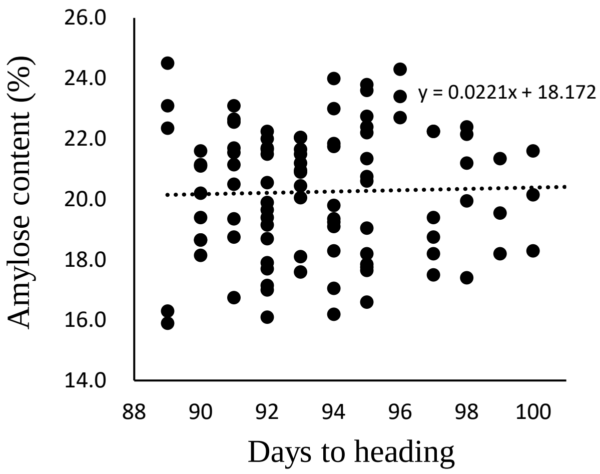

Supplemental Figure. 2. Relationship between the days to heading and amylose content in the F_5 lines
